# Status of screening and preventive efforts against diabetic kidney disease between 2013 and 2018: analysis using an administrative database from Kyoto-city, Japan

**DOI:** 10.3389/fendo.2023.1195167

**Published:** 2023-07-27

**Authors:** Yukiko Tateyama, Tomonari Shimamoto, Manako K. Uematsu, Shotaro Taniguchi, Norihiro Nishioka, Keiichi Yamamoto, Hiroshi Okada, Yoshimitsu Takahashi, Takeo Nakayama, Taku Iwami

**Affiliations:** ^1^ Department of Preventive Services, Kyoto University School of Public Health, Kyoto, Japan; ^2^ Healthtech Laboratory, Inc., Kyoto, Japan; ^3^ Miyazaki Prefectural Health Foundation, Miyazaki, Japan; ^4^ Translational Research Institute for Medical Innovation, Osaka Dental University, Hirakata, Japan; ^5^ Department of Endocrinology and Metabolism, Kyoto Prefectural University of Medicine, Graduate School of Medical Science, Kyoto, Japan; ^6^ Department of Health Informatics, Kyoto University School of Public Health, Kyoto, Japan

**Keywords:** diabetes mellitus, diabetic kidney disease, administrative database, screening test, preventive efforts against diabetic kidney disease

## Abstract

**Background:**

Japan has the second highest prevalence of dialysis use in the world. Approximately 40% of patients who begin dialysis have diabetic kidney disease (DKD). Local governments and medical facilities are required to provide preventive measures against worsening diabetes mellitus (DM). However, the percentage of patients with DM who receive such screening or interventions for DKD is unclear. This study aimed to reveal to what extent screening for DKD and preventive measures against worsening DKD are performed in patients with DM, using an administrative database in a municipality.

**Methods:**

This was a cross-sectional study that used the Kyoto-city’s administrative medical and long-term care database. Patients with a diagnosis of DM and receiving antidiabetic medication between 2013 and 2018 were defined as patients with DM and included. Patients with DKD were defined as those diagnosed with diabetic nephropathy or those with chronic kidney disease. We described the characteristics of patients with DM, diabetic complications, and extent of DKD screenings and preventive efforts against worsening of DM by fiscal year.

**Results:**

Across fiscal years, 25.8% to 27.5% of patient with DM had DKD. More than 3% of patients were on dialysis due DM in each fiscal year; approximately 15% started receiving dialysis that year. The percentage of patients who were regularly prescribed antidiabetic medication and received glycosylated hemoglobin testing ranged from 64.0% to 67.2% and from 30.6% to 36.5%, respectively. Urine microalbuminuria testing at least once a year occurred in 9.3% to 10.0%. The percentage of patients who received nutritional guidance ranged from 19.0% to 21.0%. Approximately 1% of patients received guidance for preventing DM from progressing to a disease that requires dialysis each fiscal year.

**Conclusion:**

This study from Japan, where a super-aging society has developed, using an administrative database in a municipality covering most of the elderly population clearly demonstrated an evidence-practice gap in efforts to prevent worsening of DKD. Strengthening cooperation between government and medical facilities and support for providing preventive measures against DKD are urgently needed.

## 1 Introduction

The rapid increase in the prevalence of diabetes mellitus (DM) has become a serious issue globally (1). The prevalence of DM in Japan, one of the highest aging rates countries in the world with a super-aging society, is ranked ninth in the world (1, 2). Japan has approximately 20 million patients with DM, including those with prediabetes. The number of patients with DM has been continuously increasing with changes in lifestyles and social environments (1, 3). Japan also has the second highest prevalence of dialysis use in the world (4); approximately 40% of patients who start receiving dialysis have diabetic kidney disease (DKD) as the cause of end-stage renal disease (5). Therefore, preventing the onset and worsening of DKD is urgent in terms of social and national economic burden (6).

To prevent the onset and worsening of DM, comprehensive intervention that includes tight control of blood glucose levels; control of obesity, blood pressure, and lipid levels; smoking cessation; early detection of nephropathy; and nutritional guidance is required (7, 8). Major clinical guidelines for DM recommend regular screenings for early detection of nephropathy and preventive intervention (9, 10). In response to the increase in the number of patients with DM at high risk for DKD, the Ministry of Health, Labour and Welfare of Japan developed a program to prevent worsening of diabetic nephropathy in 2016 and has been making efforts to ensure the quality of intra-regional cooperations to prevent worsening of DM (11).

However, it is unclear to what extent screenings for nephropathy are conducted and health guidance is provided to prevent disease progression in clinical practice, even they are essential for detecting DKD and preventing progression. In addition, regional gaps in the provision of these services have been identified (12). In this study, we investigated patient characteristics, complications, history of consultation with doctors, history of receiving DKD screening, and history of receiving guidance for preventing DKD progression in patients with DM, using compiled medical, screening, nursing care, and resident registry data from Kyoto-city, a Japanese metropolitan area, in order to contribute to prevent the onset and worsening of DKD.

## 2 Materials and methods

### 2.1 Study design and data sources

This was a cross-sectional study that used an administrative population-based database containing data on medical and long-term care services collected from April 2013 to March 2019 from Kyoto-city. Kyoto-city is a prefectural capital and a city designated by Japanese ordinance with a population of approximately 1.5 million and an aging rate of 28.4% (13, 14).

All citizens in Japan are enrolled in health insurance under the universal health insurance system. The database used in this study consisted of data from individuals who were enrolled in the national health insurance program (self-employed persons, retired persons, and their dependents, persons aged < 75 years) and those who were enrolled in the medical insurance system for the elderly (persons aged ≥75 years or aged 65–74 years with a disability). The database contained information on the use of medical insurance (which covers medical fees for disease or injury) and long-term care insurance (which covers fees for long-term care products and services), health checkup results, and resident registry data (15–17). It is estimated to cover approximately 35% of Kyoto-city’s population each fiscal year from 2013 to 2018. Residents aged ≥75 years were well represented; over 90% of the overall population were enrolled in the elderly’s national health insurance system for persons aged ≥75 years whereas 24.3% of persons aged <75 years were enrolled in the national health insurance system as of fiscal year 2013.

### 2.2 Participants

This study included patients with DM with available health insurance claim data in the target fiscal years (2013–2018). From a consolidated database that contained annual data between the fiscal years of 2013 and 2018, patients with a diagnosis of DM who were on antidiabetic medication were defined as patients with DM and enrolled in the study. Among these patients, those with a diagnosis of diabetic nephropathy and those with a diagnosis of chronic kidney disease were defined as patients with DKD. In this study, we described the characteristics of patients with DM, presence of diabetic complications, and extent of DKD screenings and preventive efforts against worsening of DKD performed by fiscal year. Patients whose profile data were unavailable were excluded from the study. This study enrolled both patients with type 1 DM and those with type 2 DM, which accounts for ≥95% of all cases of DM (18).

### 2.3 Variables and case definitions

YuT, TS, MKU, ST, YoT, and TI collaboratively defined the following variables through discussions: DM, kidney disease, comorbid conditions, complications, medications, and history of receiving screenings and guidance.

#### 2.3.1 Patients with DM and DKD

Patients with DM were defined as those with a diagnosis of DM (i.e., International Classification of Diseases 10th Revision code E10, E11, or E14) who had been prescribed antidiabetic medication (oral antidiabetic medication, glucagon-like peptide-1 receptor agonist, or insulin). Patients with DKD were defined as patients with DM and a diagnosis of diabetic nephropathy (ICD-10 code E102, E112, or E142) or chronic kidney disease (ICD-10 code N18 or N19), but those whose conditions were associated with acute kidney disease were excluded. The use of antidiabetic medication was defined as the use of drugs that are categorized in therapeutic category (TC) 396 and 2492 according to the TC of drugs in Japan (19). Glucagon-like peptide-1 (GLP-1) receptor agonist use was defined as the use of drugs from this category in the drug price list of Japan (20).

#### 2.3.2 Care level and comorbid conditions

Data on nursing care were from the patient’s latest care needs certification each year. There are seven levels of care needs certification, consisting of two levels of support needs certification and five levels of care needs certification, which are based on the hours of nursing care required per day (21). In this study, we categorized the nursing care levels into six levels (i.e., support needs levels 1 and 2 combined, and care needs levels 1–5). Regarding comorbid conditions, patients with hypertension were defined as those with a diagnosis of hypertension (ICD-10 code I10-15) who had been prescribed antihypertensive medication (TC 214). Patients with hyperlipidemia were defined as those with a diagnosis of hyperlipidemia (ICD-10 code E78, except for E78.3 and E78.6) who had been prescribed antihyperlipidemic medication (TC 218).

#### 2.3.3 Dialysis and complications of DM

In this study, patients with diabetic complications were defined as follows:

(1) Patients receiving dialysis: those whose health insurance claim record contains any claim codes related to the onset or implementation of dialysis.
(2) Patients that started dialysis: Among patients who meet criterion (1) above, those whose health insurance claim record contains additional codes for treatment during the initial phase of dialysis.
(3) Patients with diabetic retinopathy: those with a diagnosis of disease corresponding to ICD-10 code E103, E113, or E143.
(4) Patients with peripheral vascular disease (e.g., foot ulcer, gangrenous foot): those with a diagnosis of disease corresponding to ICD-10 code I210, I211, I212, I213, I214, or I219.
(5) Patients with acute myocardial infarction: those with a diagnosis of disease corresponding to ICD-10 code I210, I211, I212, I213, I214, or I219.
(6) Patients with stroke, cerebral infarction, or cerebral hemorrhage: those with a diagnosis of disease corresponding to ICD-10 code I610, I611, I612, I613, I614, I615, I616, I618, I619, I630, I631, I632, I633, I634, I635, I636, I638, or I639.

#### 2.3.4 Indicators of preventive efforts against DKD

In this study, the prescription of antidiabetic medication at least once every 3 months and the implementation of glycosylated hemoglobin (HbA1c) testing at least once every 3 months were used as indicators of regular medical facility visits. The extent to which DKD screening was performed was investigated in those who have not developed nephropathy based on urine microalbuminuria testing at least once a year and quantitative urine protein testing at least once a year as indicators of DKD screening. Nutritional guidance at least once a year (in outpatient, inpatient, or group settings) was used as an indicator of preventive efforts against DKD as well as an indicator of whether patients with DKD were being provided with guidance for preventing disease progression. The total number of patients who received nutritional guidance might have included duplicates if patients received guidance in multiple settings (i.e., outpatient, inpatient, and group settings). The provision of guidance to prevent progression to a disease that requires dialysis and the provision of guidance for patients with severe renal dysfunction (including those patients with end-stage renal disease) were used as indicators of whether guidance was being provided to prevent dialysis. Under the Japanese universal health insurance scheme, these types of guidance can be provided to patients with HbA1c (NGSP) ≥6.5% or those with stage II or higher diabetic nephropathy. Insurance coverage for such guidance aims to facilitate medical care by multidisciplinary medical teams to prevent DKD from progressing to a disease that requires dialysis (22).

### 2.4 Data access and cleaning methods

The authors (YuT, T.S., YoT, T.N., and T.I.) of this study had access to a database containing information on medical insurance claims, long-term care insurance claims, medical check-up results, and resident registry data. Except for patients who were excluded from the study due to missing data on their characteristics, all patients in the study were included in the descriptive analysis because there were no missing data or logically conflicting data.

### 2.5 Statistical analysis

We tabulated the number of patients with diabetic nephropathy, percentage of patients with DM who developed nephropathy, proportion of patients with various characteristics (age, sex, level of care needed), comorbid conditions, number and percentage of patients receiving dialysis, complications, number and percentage of patients who received screening testing, and number and percentage of patients who received nutritional guidance for patients with non-DKD and DKD, and preventive guidance for diabetes progression to dialysis for patients with DKD by fiscal year. Continuous data were presented as means and standard deviation and categorical data were presented as percentages. The Cochran-Armitage trend test and Jonckheere-Terpstra trend test were used to evaluate trends for patients’ characteristics and those who received screening test and guidance for preventing disease progression for DKD in the target fiscal years.

### 2.6 Ethics

This study was reviewed and approved by the Kyoto University Medical Ethics Committee (R3107). Informed consent was waived because the data was from an existing anonymized database.

## 3 Results

The number of people who had health insurance claim data in each fiscal year between 2013 and 2018 ranged from 463,877 to 497,068. The number of people who received antidiabetic medication each year ranged from 47,864 to 54,877. The number of people with a diagnosis of DM in each year ranged from 21,482 to 22,244. After excluding patients (n=8 to17) with missing data on profile data, the number of patients included in the analysis for each year ranged from 21,468 to 22,229.

### 3.1 Characteristics of patients with DM and yearly changes

**Table 1** shows the characteristics of patients with DM by fiscal year and *P* values for trend across years. Mean age increased from 73 to 75 years over 6 years. Approximately 55% of the patients with DM were male.

**Table 1:** Characteristics of patients with DM.

Number of patients (%)	FY 2013		FY 2014		FY 2015		FY 2016		FY 2017		FY 2018		*P* for trend
	N=21,468		N=21,704		N=22,212		N=21,998		N=22,229		N=22,042		
Type of diabetes													
Type 1	93	(0.4)	106	(0.5)	134	(0.6)	117	(0.5)	134	(0.6)	150	(0.7)	<0.001
Type 2	21375	(99.6)	21598	(99.5)	22078	(99.4)	21881	(99.5)	22095	(99.4)	21892	(99.3)	
Age, years													
Mean (SD)	73 (10.3)		74 (10.3)		74 (10.2)		74 (10.1)		75 (9.9)		75 (9.8)		0.024
≤60	1564	(7.3)	1492	(6.9)	1440	(6.5)	1388	(6.3)	1381	(6.2)	1303	(5.9)	Not tested
60–74	8567	(39.9)	8784	(40.5)	8878	(40.0)	8377	(38.1)	8204	(36.9)	7807	(35.4)	Not tested
≥75	11337	(52.8)	11428	(52.7)	11894	(53.5)	12233	(55.6)	12644	(56.9)	12932	(58.7)	Not tested
Male sex	11583	(54.0)	11843	(54.6)	12128	(54.6)	12049	(54.8)	12206	(54.9)	12093	(54.9)	0.042
Level of care needed													
Certified	4678	(21.8)	5380	(24.8)	5682	(25.6)	5788	(26.3)	6016	(27.1)	5175	(23.5)	<0.001
[Level]													
Requiring help	1443	(30.8)	1565	(29.1)	1677	(29.5)	1673	(28.9)	1663	(27.6)	1053	(20.3)	Not tested
Level 1	746	(15.9)	817	(15.2)	885	(15.6)	907	(15.7)	992	(16.5)	844	(16.3)	Not tested
Level 2	879	(18.8)	1036	(19.3)	1063	(18.7)	1103	(19.1)	1150	(19.1)	1058	(20.4)	Not tested
Level 3	635	(13.6)	704	(13.1)	769	(13.5)	783	(13.5)	786	(13.1)	795	(15.4)	Not tested
Level 4	545	(11.7)	653	(12.1)	704	(12.4)	723	(12.5)	832	(13.8)	775	(15.0)	Not tested
Level 5	430	(9.2)	605	(11.2)	584	(10.3)	599	(10.3)	593	(9.9)	650	(12.6)	Not tested
Comorbid conditions													
Hypertension with medication use	12650	(58.9)	12679	(58.4)	13019	(58.6)	12939	(58.8)	12948	(58.2)	12720	(57.7)	0.022
Hyperlipidemia with medication use	10902	(50.8)	11149	(51.4)	11628	(52.4)	11668	(53.0)	11693	(52.6)	11844	(53.7)	<0.001

FY, fiscal year.

Type 2 includes unspecified diabetes mellitus.

**Table 2** shows the number and percentage of patients with DM who were receiving dialysis, had diabetic complications, or regularly visited a medical facility by fiscal year. Among all patients with DM, the number of patients who were receiving dialysis ranged from 708 to 847 (3.3% to 3.8%) throughout the target fiscal years. There were ≥100 patients (13.8% to 16.9% of all patients receiving dialysis) who started receiving dialysis in each fiscal year. The percentage of patients who were prescribed antidiabetic medication at least once every 3 months, an indicator of regular medical facility visits, increased slightly during the study period, from 64.0% to 67.2%. The percentage of patients who received HbA1c testing at least once every 3 months ranged from 30.6% to 36.5%.

**Table 2:** Number and percentage of patients with DM on dialysis, with diabetic complications, or regularly visiting medical facilities.

Number of patients (%)	FY 2013		FY 2014		FY 2015		FY 2016		FY 2017		FY 2018		*P* for trend
	N=21,468		N=21,704		N=22,212		N=21,998		N=22,229		N=22,042		
Diabetic complication													
Dialysis	708	(3.3)	776	(3.6)	847	(3.8)	805	(3.7)	830	(3.7)	786	(3.6)	0.121
Initiated during that year (among patients with dialysis)	120	(16.9)	120	(15.5)	131	(15.5)	111	(13.8)	138	(16.6)	119	(15.1)	0.548
Diabetic kidney disease	5762	(26.8)	5627	(25.9)	5729	(25.8)	5906	(26.8)	5773	(26.0)	6052	(27.5)	0.083
Diabetic retinopathy	5278	(24.6)	5470	(25.2)	5668	(25.5)	5580	(25.4)	5556	(25.0)	5555	(25.2)	Not tested
Diabetic neuropathy	2026	(9.4)	1923	(8.9)	1922	(8.7)	1934	(8.8)	1850	(8.3)	1704	(7.7)	Not tested
Diabetic foot ulcer	625	(2.9)	677	(3.1)	689	(3.1)	716	(3.3)	720	(3.2)	721	(3.3)	Not tested
Acute myocardial infarction	1703	(7.9)	1691	(7.8)	1815	(8.2)	1867	(8.5)	1743	(7.8)	1786	(8.1)	Not tested
Stroke	5376	(25.0)	5400	(24.9)	5544	(25.0)	5302	(24.1)	4794	(21.6)	4703	(21.3)	Not tested
Regular medical facility visits													
Prescribed antidiabetic medication [at least once per 3 months]	13748	(64.0)	13893	(64.0)	14641	(65.9)	14332	(65.2)	14847	(66.8)	14810	(67.2)	<0.001
HbA1c testing [at least once per 3 months]	7132	(33.2)	6958	(32.1)	6959	(31.3)	6737	(30.6)	7981	(35.9)	8051	(36.5)	<0.001

DM, diabetes mellitus; FY, fiscal year; HbA1c, glycosylated hemoglobin.

### 3.2 Screening testing and preventive guidance for DKD

**Table 3** shows the number and percentage of patients who had received nephropathy screening testing and received nutritional guidance at a medical facility among those with DM but did not have DKD by fiscal year. The percentage of patients who underwent urine microalbuminuria testing, which is important for detecting DKD, at least once a year ranged from 9.3% to 10.0%. Similarly, the percentage who underwent quantitative urine protein testing at least once a year ranged from 6.3% to 7.2%. The percentage of patients who received nutritional guidance ranged from 19.0% to 21.0%, with slight increases each fiscal year.

**Table 3:** Number and percentage of patients received screening testing or nutritional guidance among non-DKD patients.

Number of patients (%)	FY 2013		FY 2014		FY 2015		FY 2016		FY 2017		FY 2018		*P* for trend
	n=15,706		n=16,077		n=16,483		n=16,092		n=16,456		n=15,990		
Screening testing													
Urine protein, quantitative [at least once per year]	1012	(6.4)	1010	(6.3)	1047	(6.4)	1067	(6.6)	1106	(6.7)	1153	(7.2)	<0.001
Urine microalbuminuria [at least once per year]	1533	(9.8)	1509	(9.4)	1649	(10.0)	1492	(9.3)	1619	(9.8)	1529	(9.6)	0.617
Serum creatinine [at least once per year]	14713	(93.7)	15066	(93.7)	15544	(94.3)	15223	(94.6)	15530	(94.4)	15026	(94.0)	0.016
Nutritional guidance (Any)	3020	(19.2)	3064	(19.1)	3137	(19.0)	3091	(19.2)	3280	(20.4)	3374	(21.0)	<0.001
Outpatient	682	(4.3)	666	(4.1)	692	(4.2)	641	(4.0)	681	(4.2)	725	(4.5)	Not tested
Inpatient	2058	(13.1)	2086	(13.0)	2128	(12.9)	2189	(13.6)	2291	(14.2)	2388	(14.8)	Not tested
Group	280	(1.8)	312	(1.9)	317	(1.9)	261	(1.6)	308	(1.9)	261	(1.6)	Not tested

DM, diabetes mellitus; FY, fiscal year.

### 3.3 Preventive guidance for diabetes progression to dialysis for patients with DKD

**Table 4** shows the number and percentage of patients who received “nutritional guidance” and “preventive guidance for diabetes progression to dialysis” among those with DKD. The total number and percentage of patients who received nutritional guidance in the outpatient (individual), inpatient (individual), and inpatient or outpatient (group) settings ranged from 42.7% to 46.3%, with slight decreases each fiscal year. Approximately 1% of patients received guidance for preventing DM from progressing to a disease that requires dialysis throughout the target fiscal years.

**Table 4:** Number and percentage who received nutritional guidance or preventive guidance for diabetes progression to dialysis among DKD patients.

Number of patients (%)	FY 2013		FY 2014		FY 2015		FY 2016		FY 2017		FY 2018		*P* for trend
	n=5,762		n=5,627		n=5,729		n=5,906		n=5,773		n=6,052		
Nutritional guidance (Any)^*^	2652	(46.0)	2626	(46.3)	2586	(44.8)	2587	(43.8)	2518	(43.6)	2585	(42.7)	<0.001
Outpatient	748	(13.0)	706	(12.5)	669	(11.7)	636	(10.8)	640	(11.1)	712	(11.8)	Not tested
Inpatient	1570	(27.2)	1591	(27.9)	1587	(27.4)	1661	(28.1)	1603	(27.8)	1609	(26.6)	Not tested
Group	334	(5.8)	329	(5.8)	330	(5.8)	290	(4.9)	275	(4.8)	264	(4.4)	Not tested
Guidance for prevention of dialysis													
Guidance for preventing diabetes from progressing to a disease that requires dialysis	64	(1.1)	62	(1.1)	38	(0.7)	54	(0.9)	62	(1.1)	69	(1.1)	0.750
Guidance for patients with severe renal dysfunction (including those patients with end-stage renal disease)	−		−		−		0	(0.0)	2	(0.0)	0	(0.0)	Not tested

*Values might include duplicates if patients received guidance in multiple settings, which were counted separately.

Guidance for patients with severe renal dysfunction has been covered by health insurance since 2016.

Fees for providing guidance to patients with renal failure has been claimed as fees for providing guidance to patients with severe renal dysfunction since 2018.

DM, diabetes mellitus; FY, fiscal year.

## 4 Discussion

Using an administrative, population-based database that contains comprehensive medical and long-term care data in a metropolitan area in Japan, we investigated data on characteristics of patients with DM and the extent of screenings and preventive measures against disease progression across years. In this study, we identified some issues in preventive measures currently being used against worsening of DM, low implementation rates of DKD screening. This study is expected to contribute to the prevention of DM progression by revealing the status and challenges in Japan, a country that is experiencing super-aging society and greater medical and social burdens due to the increase in the prevalence of DM and DKD ahead of other countries.

### 4.1 Current status of access to healthcare services for DM

Although appropriate glycemic control is basic care for preventing the development and worsening of DKD (7, 18), the present study revealed that only two-thirds of patients with DM were regularly prescribed antidiabetic medication and only one-third of patients with DM regularly received HbA1c testing, indicating that many patients with DM might have not enough regular visits to outpatient clinics. A study reported that longer duration of receiving treatment is associated with lower drug adherence rates (23). Previous studies in Japan demonstrated that the percentage of patients with DM who regularly received HbA1c testing was ≥60% (12, 24, 25), whereas it was ≤50% in the present study. The present study contained more elderly people, therefore the findings from our study might suggest the difficulty for elderly people to regularly visit outpatient clinics (24) and issues in preventive measures against worsening of DM in a rapidly aging society, including poor drug adherence and accessibility of medical services. In addition, the complexity of the prescription including polypharmacy among DM patients with comorbidity might also influence to lower drug adherence and regularly prescribed medication.

### 4.2 Low screening and implementation rates for preventive measures against worsening of DKD

This study showed low implementation rates for quantitative urine albumin and protein testing as DKD screening, which are recommended in the guidelines (7, 8). Although urine screening testing is recommended every 3 to 6 months and is covered by health insurance in Japan, the actual frequency of testing is lower than recommended (23, 25, 26). The actual frequency of urine screening testing in Japan is lower than that in other developed countries (27). The burden of ordering the screening test in the usual practice might be one of the possible explanations. Particularly, low implementation rates among non-DKD patients, where early screening is critical, are noteworthy. Therefore, it is necessary to encourage healthcare professionals to have patients with DM regularly undergo urine screening testing and further studies to investigate the reason of low screening rate is needed.

The present study also revealed that sufficient intervention was not provided to allow for early diagnosis and prevent DKD from progressing to end-stage renal disease. In Japan, the fee for providing guidance to prevent DM from progressing to a disease that requires dialysis has been covered by health insurance since 2012. However, among the target population of this study, the number of patients who received such guidance remained low throughout the target years. One possible reason is that only a limited number of medical facilities met the requirements for providing this guidance under the health insurance program, i.e., having a multidisciplinary medical team to prevent disease progression and having dedicated staff who have experience in providing guidance on DM control. A previous study reported that one reason why guidance was not provided was the lack of capability in medical facilities, such as unavailability of staff (28). Another possibility is that the insurance of providing this guidance might not be enough to cover its actual cost of implementation at the general practitioner level. Therefore, it is necessary to train professionals who can provide nutritional guidance, review the requirements and insurance system for medical facilities to provide preventive guidance for diabetes progression to dialysis, and strengthen partnerships between medical facilities that meet the requirements and those that do not meet the requirements in each community in order to improve access to prevention guidance for patients with DM and at high risk for dialysis.

When a program to prevent worsening of DKD was developed in 2016, the Ministry of Health, Labour and Welfare specified basic activities for the program according to the Plan–Do–Check–Act cycle, including the identification of patients at high risk of worsening DKD, cooperation with stakeholders, and program review. Under this framework, local governments have been making efforts to prevent DM progression to the need for dialysis through improving access to healthcare services for patients with DM who have not visited a medical facility and those who have stopped visiting medical facilities as well as providing health guidance to these patients in cooperation with medical facilities (11). In the present study, the percentage of patients who regularly received antidiabetic medication and regularly receive HbA1c testing slightly increased after 2017, respectively, but remained low. Therefore, more active and enough quality of implementation is needed (12, 29).

### 4.3 Future perspectives

Through the analysis of a database that covers much of the population aged ≥75 years, this study revealed an evidence-practice gap in efforts to prevent the onset and worsening of DKD, including a low DKD screening and implementation rates for prevention interventions. Close investigation of why evidence has not been adequately applied in practice and strengthening cooperation between the government and medical facilities is urgently needed, as well as providing support for disseminating health guidance in order to promote early detection of and preventive intervention for DKD progression. In addition, healthcare professionals should be informed of the low implementation rate of screening testing for early detection of DKD. Moreover, enabling more medical facilities to provide preventive guidance for diabetes progression to dialysis regardless of their size, by allocating dedicated healthcare professionals such as nutritionists and revising the extent of health insurance coverage, should be considered.

To encourage patients to manage their lifestyle and control blood glucose levels, it is important to promptly put in place an environment that allows the use of digital technology that enables healthcare professionals to provide patients with high-quality medical services by linking personal health records (various health records generated in the lives of individuals) to electronic health records (medical records from multiple medical facilities, including personal information).

The management of DM and interventions for preventing DKD and its progression are expected to be more complicated due to aging of the society. It is important to take measures against lifestyle-related diseases that are suitable for a super-aging society, including strengthening efforts to prevent worsening of DM in elderly patients and maintaining their quality of life and activities of daily living and an adequate support for ICT use.

## 5 Limitations

This study has some limitations. Under the Japanese health insurance system, it might not be possible to collect accurate data on people who repeatedly enroll and withdraw from the national health insurance system. The database used in this study was dominated by data from self-employed persons or unemployed persons, their family members, and persons aged ≥75 years. Therefore, care should be exercised in generalizing the results of this study, including regular medical facility visits or prescriptions among elderly people who tend to admit to medical and nursing care facilities. This study was based on health insurance claim data. Therefore, there were limitations in identifying whether patients had been diagnosed with DM or diabetic nephropathy, numbers of patients, types of complications, and screening and guidance (30–32).

## 6 Conclusions

This study performed in Japan, where a super-aging society has developed and one of the highest proportions of older population in the world, using an administrative database in a municipality covering most of the elderly population clearly demonstrated an evidence-practice gap in efforts to prevent worsening of DKD. Strengthening cooperation between governments and medical facilities and support for providing measures to prevent DKD are urgently needed.

## Data availability statement

Data analyzed in this study are not publicly available and cannot be shared with external researchers due to restrictions on access to the data by Kyoto City.

## Ethics statement

The studies involving human participants were reviewed and approved by the Kyoto University Medical Ethics Committee (R3107). Written informed consent from the patients/participants or patients/participants’ legal guardian/next of kin was not required to participate in this study in accordance with the national legislation and the institutional requirements.

## Author contributions

Concept and design: YuT, TS, MKU, KY, YoT, TI. Acquisition of data: YoT, TN, TI. Analysis and interpretation of data: YuT, TS, ST, NN, HO, KY, YoT, TI. Drafting of the manuscript: YuT, TS, MKU, ST, NN, HO, KY, YoT, TN, TI. Statistical analysis: YuT, TS. Provision of study materials or patients: YoT, TN, TI. Obtaining funding: YoT, TN, TI. Administrative, technical, or logistic support: TN, TI. Supervision: TN, TI. All authors contributed to the article and approved the submitted version.
